# Individualized dynamic ablation strategy for persistent atrial fibrillation based on ibutilide response

**DOI:** 10.1186/s43044-026-00745-4

**Published:** 2026-05-13

**Authors:** Jing Hu, Yu Xia, Yu Tao, Le Li, Xu Meng, Min Lin, Yin Liu, Hengli Lai, Ligang Ding, Yan Yao

**Affiliations:** 1 Jiangxi Provincial People’s Hospital，The First Affiliated Hospital of Nanchang Medical College, Nanchang, China; 2 National Center for Cardiovascular Diseases and Fuwai Hospital, Chinese Academy of Medical Sciences and Peking Union Medical College, Beijing, China; 3 The University of Hong Kong-Shenzhen Hospital, Shenzhen, China; 4 Tianjin First Central Hospital, Tianjin, China

**Keywords:** Persistent atrial fibrillation, Radiofrequency ablation, Ibutilide, Recurrence predictive factors

## Abstract

**Background:**

Persistent atrial fibrillation (PsAF) ablation faces a dilemma between insufficient pulmonary vein isolation (PVI) leading to high recurrence and excessive ablation increasing complications without clear benefit. Ibutilide may help identify critical ablation targets by modifying the arrhythmia substrate.

**Methods:**

This retrospective single-center study analyzed 367 PsAF patients undergoing ibutilide-guided ablation. After PVI, patients converting directly to sinus rhythm (SR) (PVI group, *n* = 86) received no further ablation. Non-responders (PVI+Linear group, *n* = 281) underwent linear ablation and were categorized by acute termination pattern: Directly to SR (*n* = 109), AFL to SR (*n* = 87), DC to SR (*n* = 75), or Failure (*n* = 10). Subgroups based on low-voltage areas (LVZs) and AF duration were analyzed. AF recurrence (> 30s post 3-month blanking period) was assessed.

**Results:**

Over a mean follow-up, AF-free survival rate was non-significantly higher in the PVI group vs. PVI+Linear group ((73.9% vs. 60.7%, *P* = 0.070). In the PVI+Linear group, success rates differed significantly by acute termination pattern (Direct to SR:67.8%, AFL to SR:72.6%, DC to SR:26.4%, Failure to SR:30.0%, *P* < 0.001), LVZs presence (Absent:63.8% vs. Present:55.8%, *P* < 0.001), and AF duration (≤ 1 year:73.9%, 1–5 year:55.3%, ≥ 5 year:44.6%, *P* < 0.001). Female, larger LA diameter, longer AF duration, and DC/Failure termination patterns independently predicted recurrence.

**Conclusion:**

The use of ibutilide may help identify “PVI‑sensitive” atrial fibrillation, reducing the extent of ablation without compromising efficacy.Female sex, LA enlargement, prolonged AF duration, and requiring DC/failing to terminate acutely predict recurrence.

**Graphical Abstract:**

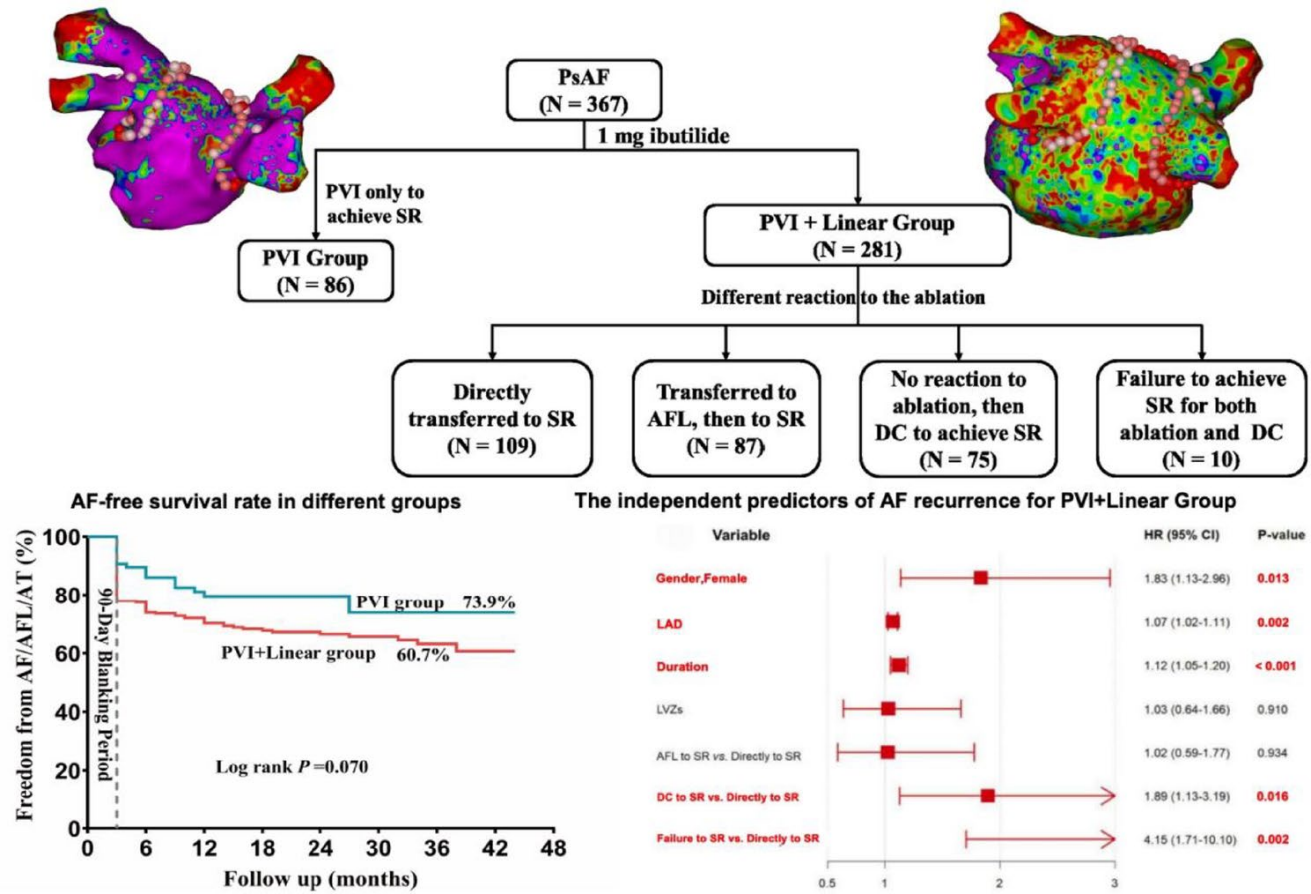

## Introduction

Atrial fibrillation (AF) is one of the most common arrhythmias in clinical practice [1]. Persistent atrial fibrillation(PsAF), characterized by complex electrophysiological and structural remodeling mechanisms, remains controversial in terms of ablation strategies [23]. While traditional ablation primarily relies on pulmonary vein isolation (PVI), PVI alone proves insufficient for PsAF, often necessitating empirical linear ablation or substrate modification [4–8]. However, two core contradictions persist. First, excessive ablation prolongs procedural time and increases risk of complications (e.g. pericardial tamponade [9], atrial-esophageal fistula and phrenic nerve injury [10]), and it has failed to demonstrate clear clinical advantages [8, 11–16]. Second, insufficient ablation fails to eliminate non-pulmonary vein triggers or drivers as maintainers of AF, resulting in high recurrence rates [17]. The FLOW-AF study found that PVI plus substrate ablation improved AF-free survival by an absolute 51% compared to PVI alone [18]. Additionally, studies such as VENUS [7], PROMPT-AF [8], STABLE-SR-II [16], and TAILORED-AF [19] have demonstrated that a “PVI-plus” strategy significantly lowers the recurrence rate after ablation. In patients with AF complicated by heart failure, inadequate ablation accelerates left atrial (LA) remodeling [20]. This dilemma stems from the lack of effective tools for real-time intraoperative assessment of arrhythmogenic substrates, forcing ablation strategies into an impasse between “empirical expansion ablation” and “conservative observation ablation”.

Ibutilide, a Class III antiarrhythmic drug, blocks the rapid delayed rectifier potassium current to prolong atrial effective refractory periods. By promoting AF wavefront fusion and reducing complex electrogram distribution, it exposes critical ablation targets. [13]This study proposes an personalized dynamic ablation strategy based on intraoperative ibutilide response. By unmasking latent substrate, ibutilide may facilitate the distinction between PV-dependent and non-PV-dependent mechanisms; however, this study was not designed to prove causality in the absence of a non-ibutilide control arm. This strategy integrated drug response with anatomical ablation pathways, establishing a tripartite individualized treatment framework of “drug-electrophysiology-anatomy” to reduce unnecessary ablation and associated complications.

This study aimed to develop an individualized dynamic ablation strategy for persistent AF based on intraoperative ibutilide response and evaluate its clinical efficacy.

## Method

### Study population

This study retrospectively analyzed 367 patients with PsAF who underwent ibutilide-guided radiofrequency ablation at Fuwai Hospital(Beijing, China) between April 2021 and June 2024. Eleven cases lost to follow-up were excluded. Written informed consent was obtained from all patients or their legally authorized representatives prior to study inclusion. The study was approved by the Ethics Committee of Fuwai Hospital (Approval No. 20240576).

### Study procedures and follow-up

Ibutilide Administration and Dynamic Ablation Strategy: Following disinfection, draping, and local anesthesia, femoral venous puncture was performed. A long sheath was introduced via the femoral vein, followed by transseptal puncture. The mapping catheter(Pentaray, Biosense-Webster) was then advanced into the LA. After LA anatomy and voltage mapping, 1 mg ibutilide fumarate was administered intravenously with continuous rhythm monitoring. PVI was simultaneously performed using a contact force ablation catheter༈ThermoCool SmartTouch SF, Biosense-Webster༉༈Lesion index (Visitag) 400 for posterior and 500 for anterior wall༉.

**Fig. 1:**
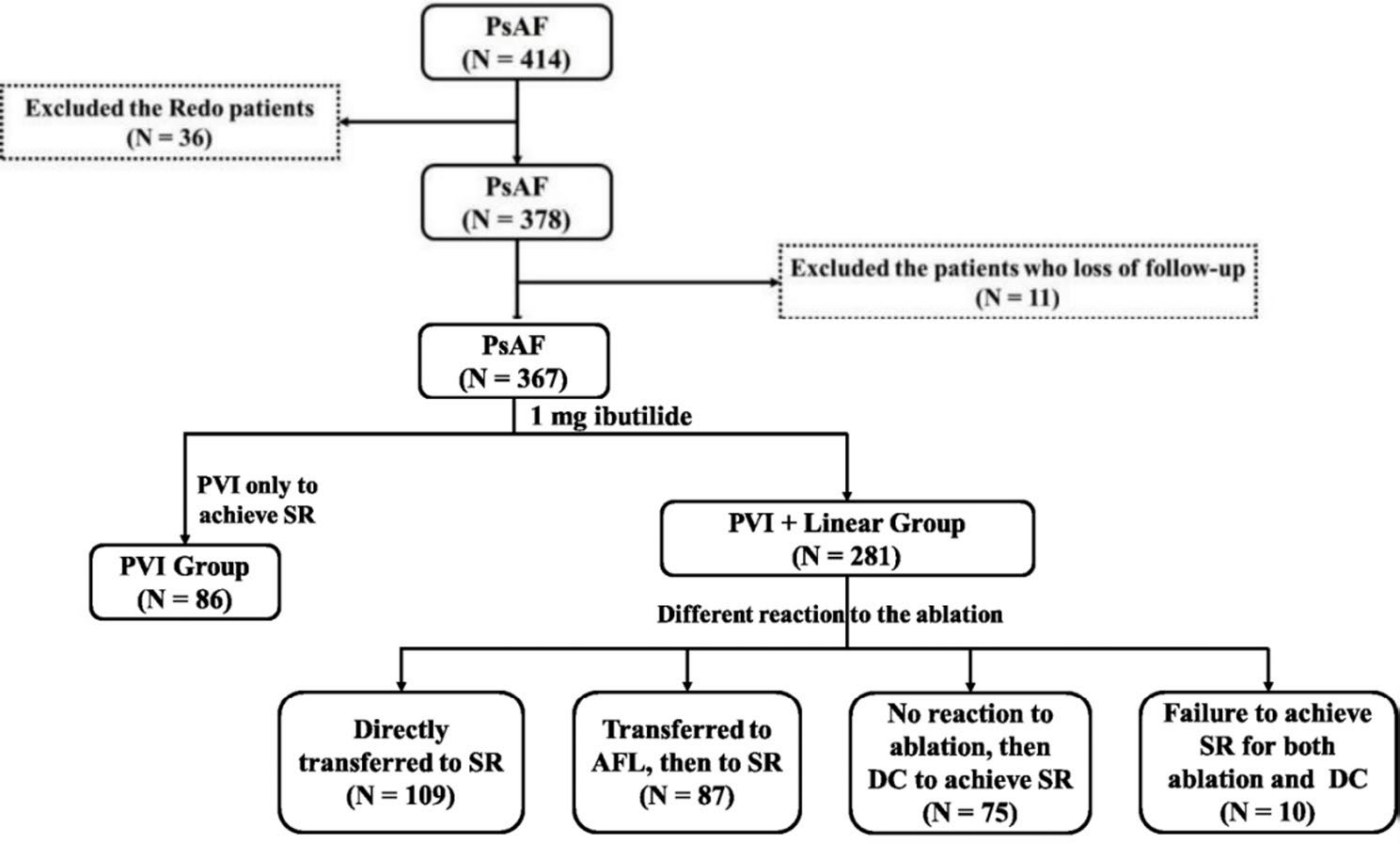
Study population flowchart. *Note.* AFL: Atrial flutter; DC: Direct-electrical cardioversion; PsAF: Persistent atrial fibrillation; PVI: Pulmonary vein isolation; SR: Sinus rhythm

After completion of PVI, patients were categorized into two groups based on their response and underwent individualized ablation according to a dynamic ablation strategy: 1. Sinus rhythm (SR)conversion (AF directly converted to SR): This group underwent PVI only and was classified as the PVI group. 2.The remaining patients were assigned to the PVI + linear group and underwent linear ablation(including the roof line/mitral valve isthmus line/interatrial septal isthmus line/anterior wall line/tricuspid valve isthmus line). Bidirectional block was confirmed by differential pacing along the line and absence of ≥ 100 ms conduction delay on the opposite side. According to the different acute termination status of linear ablation, it was divided into four subgroups (showed as Fig. 1): (1) Directly to SR (AF directly transferred to SR; *N* = 109); (2) Atrial flutter (AFL) to SR (AF transferred to AFL, then to SR; *N* = 87): High-density mapping was performed to identify and ablate the critical isthmus; (3) Direct-electrical cardioversion (DC) to SR (No reaction to ablation, then DC to achieve SR; *N* = 75); (4)Failure to SR (Failure to achieve SR for both ablation and DC; *N* = 10)(Among them, three patients converted to sinus rhythm within the 3-month blanking period.).

Additionally, the PVI + linear group was stratified into subgroups based on two criteria: (1) the presence of low-voltage areas (defined as bipolar voltage ≤ 0.5 mV during AF and occupying > 5% of the LA surface area) [21], and (2) AF duration(from first documented episode) of ≤ 1 year, 1–5 years, and ≥ 5 years, for separate analyses.

After the procedure, patients received novel oral anticoagulants and the antiarrhythmic drug amiodarone hydrochloride (avoided in patients with bradycardia) for three months. Anticoagulation was continued beyond this period based on the CHA₂DS₂-VASc score. During follow-up(6 months to 44 months), occurrences of AF recurrence, stroke, heart failure, and death were documented. AF recurrence was defined as AF, AFL, or atrial tachycardia (AT) lasting more than 30 s after the 3-month blanking period [2].

### Statistical analysis

Continuous variables were presented as mean ± standard deviation (SD) or median (interquartile range), and categorical variables were presented as percentages. Recurrence-free survival was analyzed using Kaplan-Meier curves, with between-group comparisons performed using the log-rank test. Risk factors for AF recurrence were identified using Cox proportional hazards regression models. A *P*-value less than 0.05 was considered statistically significant for all two-tailed tests. All statistical analyses were conducted using R software (version 3.6.0; R Foundation for Statistical Computing, Vienna, Austria).

## Results

### Baseline characteristics

Table 1 showed the detail characteristics of the patients at baseline. A total of 367 patients were enrolled, with a mean age of 59.9 ± 9.8 years. Females comprised 20.2% of the cohort, while low-voltage areas in the LA were present in 23.7% of patients. Among them, 86 patients were assigned to the PVI group and 281 to the PVI + linear group. A statistically significant difference in the prevalence of low-voltage areas was observed between the two groups (4.7% vs. 29.5%, *P* < 0.001), whereas no significant differences were found in other baseline characteristics.

**Table 1:** Baseline characteristics of all patients

Age (years)	Total (*N* = 367)	PVI Group (*n* = 86)	PVI+Linear Group (*n* = 281)	*P*-value
	59.9 ± 9.8	58.4 ± 9.0	60.4 ± 10.0	0.099
Gender, Female	74 (20.2%)	14 (16.3%)	60 (21.4%)	0.383
Hypertension	172 (46.9%)	35 (40.7%)	137 (48.8%)	0.235
Diabetes	53 (14.4%)	13 (15.1%)	40 (14.2%)	0.978
Stroke	45 (12.3%)	10 (11.6%)	35 (12.5%)	0.987
Coronary heart disease	50 (13.6%)	14 (16.3%)	36 (12.8%)	0.522
Hyperlipidemia	98 (26.7%)	23 (26.7%)	75 (26.7%)	1
CHA_2_DS_2_-VASc score	1 (0, 2)	1 (0, 2)	1 (0, 3)	0.296
HASBLED score	0 (0, 1)	0 (0, 1)	0 (0, 1)	0.842
Left atrial diameter (mm)	43.0 ± 4.8	43.0 ± 3.5	43.0 ± 5.1	0.973
Left ventricular end-diastolic diameter (mm)	48.0 ± 4.0	48.2 ± 4.0	47.9 ± 4.0	0.597
Left ventricular ejection fraction (%)	63.0 ± 4.6	63.2 ± 4.0	63.0 ± 4.8	0.728
NT-proBNP (pg/ml)	735 (434, 804)	763 (561, 810)	712 (397, 799)	0.127
Atrial fibrillation duration (years)	2.0 (0.5, 4.0)	2.0 (0.5, 4.0)	2.0 (0.5, 3.0)	0.482
Low-voltage area	87 (23.7%)	4 (4.7%)	83 (29.5%)	< 0.001

Note. Ibutilide was administered at the start of surgery to both the PVI group and the PVI+linear group

### Follow-up data

**Fig. 2:**
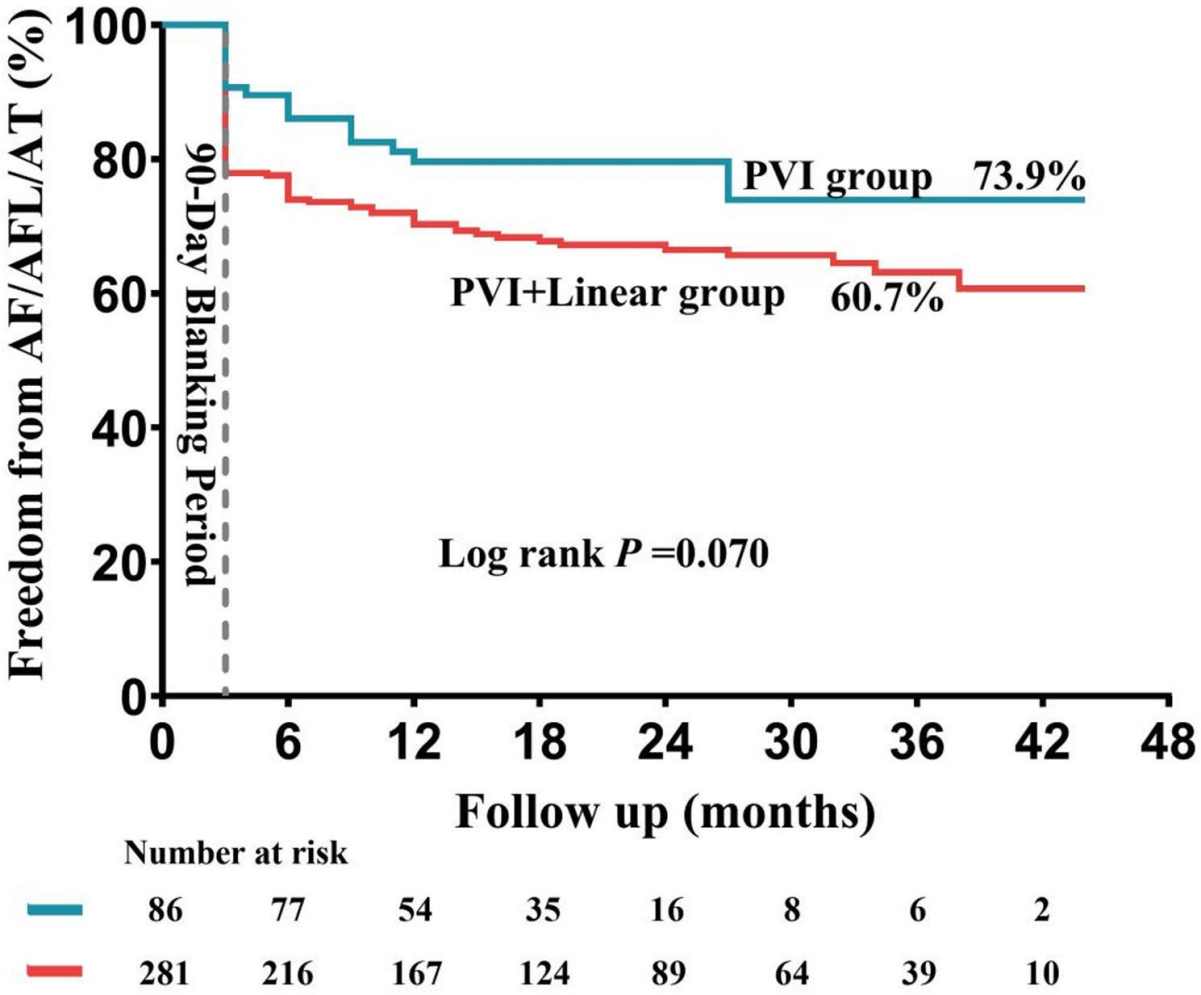
The Kaplan-Meier curve of success rates in different groups. Note. Ibutilide was administered at the start of surgery to both the PVI group and the PVI+linear group.AF: Atrial fibrillation; AT: Atrial tachycardia; Else abbreviations: Same as Fig. 1

**Fig. 3:**
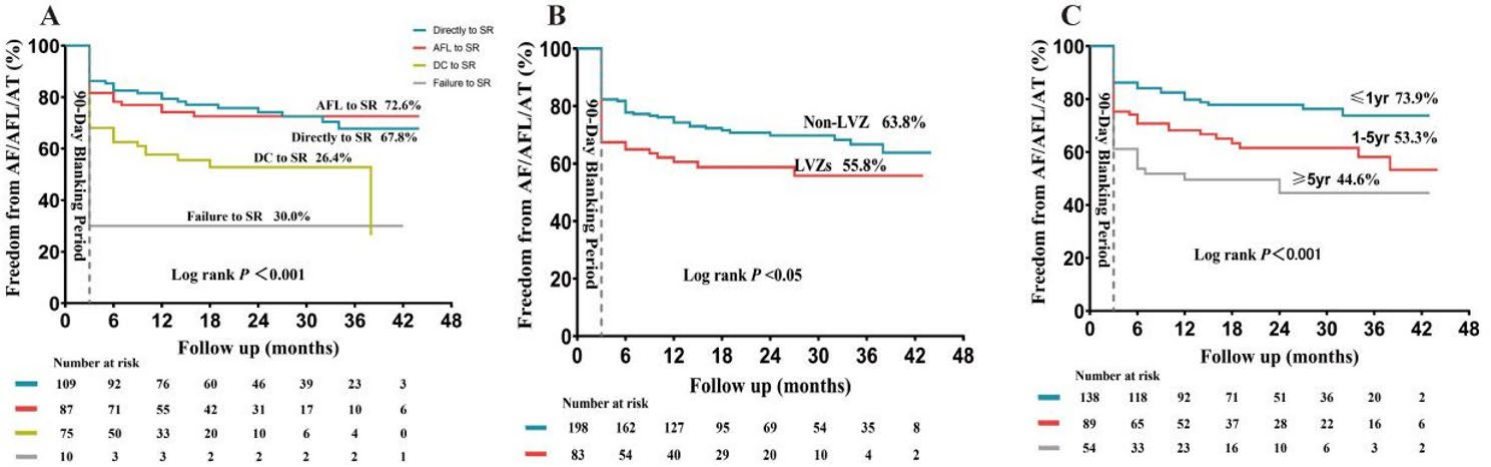
The Kaplan-Meier curves of success rates in different subgroups with PVI+Linear Group.A. The termination patterns of AF; B. The LVZs; C. The duration of AF (≤ 1 year / 1–5 years /≥5 years). Note LVZs: Low-voltage area; Else abbreviations: Same as Fig. 1 and 2

The PVI group demonstrated marginally higher AF-free survival rate compared to the PVI+linear group (73.9% vs. 60.7%), though this difference was not statistically significant (log-rank *P* = 0.070; Fig. 2). Within the PVI + linear group, however, significant differences in AF-free survival emerged across acute termination patterns (Directly to SR vs. AFL to SR vs. DC to SR vs. Failure to SR: 67.8% vs. 72.6% vs. 26.4% vs. 30.0%, Log rank *P* < 0.001; Fig. 3A), LVZ status (Present vs. Absent: 55.8% vs. 63.8%, log-rank *P* < 0.001; Fig. 3B), and AF duration subgroups (≤ 1 year vs. 1–5 years vs. ≥5 years: 73.9% vs. 55.3% vs. 44.6%, Log rank *P* < 0.001; Fig. 3C).

### Complications

The PVI group experienced 1 case (1.16%) of polymorphic ventricular tachycardia without other severe complications(It occurred within 3 min after the ibutilide, was terminated with magnesium sulfate cardioversion in < 15 s, and was considered ibutilide-related rather than ablation-induced.). Major complications in the PVI+linear group comprised polymorphic ventricular tachycardia (*N* = 1, 0.36%), pericardial tamponade (*N* = 1, 0.36%), and acute cerebral infarction (*N* = 1, 0.36%). Additional events included LA appendage disconnection (*N* = 1, 0.36%) and transient gastroparesis (*N* = 3, 1.07%).

### Risk factors for recurrence

**Fig. 4:**
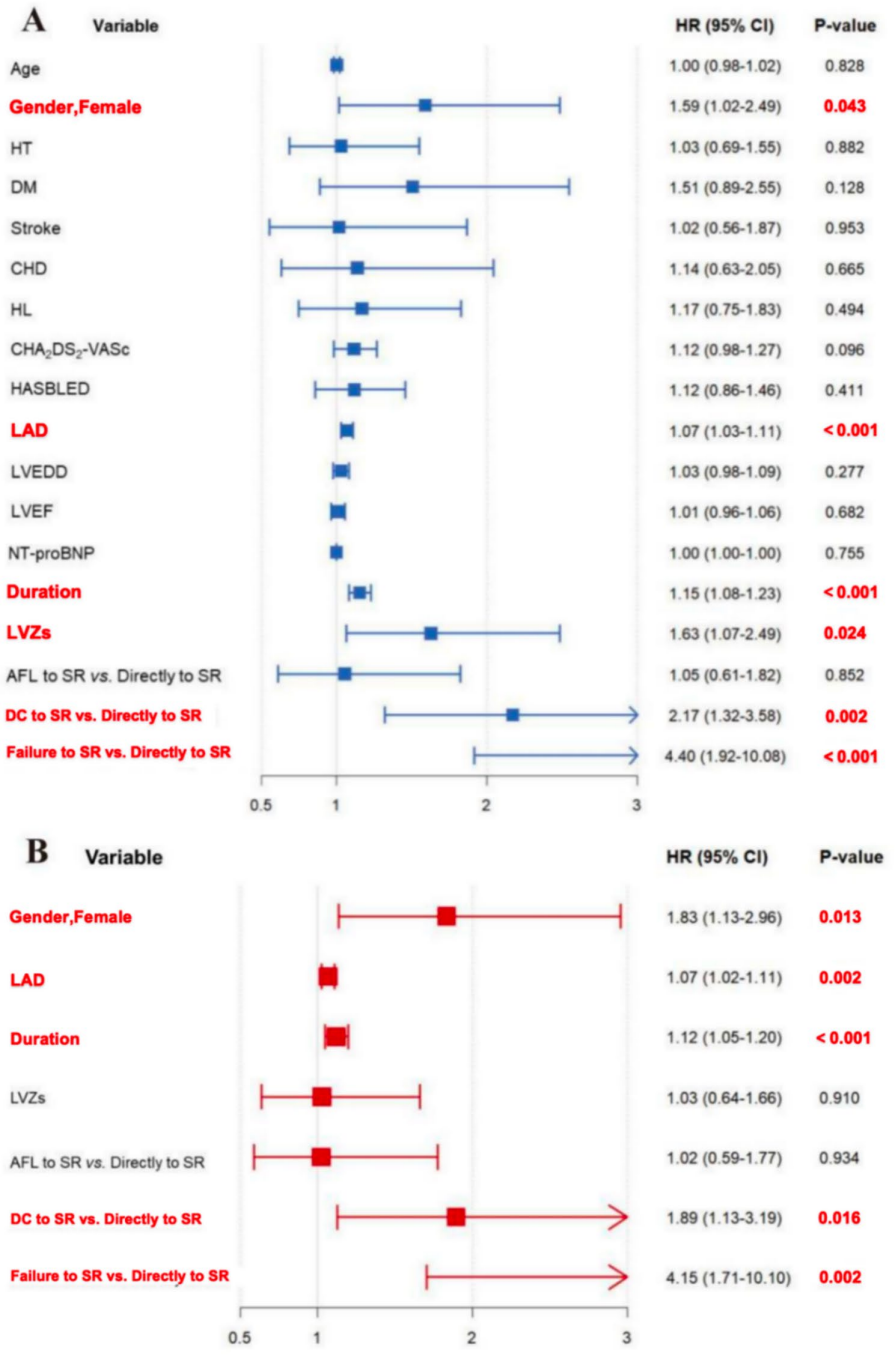
Univariate (**A**) and multivariate (**B**) Cox regression analysis of forest plots. Forest plots displaying HRs and 95% CIs of AF recurrence in the PVI + Linear group. Variables with *P* < 0.05 included in the univariate analysis were included for multivariate analysis to determinate the independent predictors of AF recurrence. HT: Hypertension; DM: Diabetes; CHD: Coronary heart disease; HL: Hyperlipidemia; LAD: Left atrial diameter; LVEDD: Left ventricular end-diastolic diameter; LVEF: Left ventricular ejection fraction. Else abbreviations: Same as Fig. 1, 2 and 3

As showed in Fig. 4A, univariate Cox regression analysis indicated that female (HR 1.59, 95% CI 1.02 − 2.49, *P* = 0.043), LA diameter (HR 1.07, 95% CI: 1.03 − 1.11, *P* < 0.001), duration (HR 1.15, 95% CI 1.08–1.23; *P* < 0.001), LVZs (HR 1.63, 95% CI 1.07–2.49; *P* = 0.024) and acute termination patterns (DC to SR vs. Directly to SR: HR 2.17, 95% CI: 1.32 − 3.58, *P* = 0.002; Failure to SR vs. Directly to SR: HR 4.40, 95% CI: 1.92 − 10.08, *P* < 0.001) were significantly associated with the increased risk of AF recurrence. To further confirm the independent prediction of these variables, only significant variables in univariate Cox regression analysis were included for stepwise multivariate analysis. Multivariate Cox regression analysis showed that female (HR 1.83, 95% CI 1.13–2.96, *P* = 0.013), LA diameter (HR 1.07, 95% CI 1.02–1.11, *P* = 0.002), duration (HR 1.12, 95% CI 1.05–1.20; *P* < 0.001), DC termination patterns (HR 1.89, 95% CI 1.13–3.19, *P* = 0.016) and failure termination patterns (HR 4.15, 95% CI 1.71–10.10, *P* = 0.002) were significantly and independently associated with the increased risk of AF recurrence after adjusting for LVZs (Fig. 4B).

## Discussion

### Dilemmas of ablation strategies for PsAF and the shift to dynamic ablation strategy

A fundamental challenge in catheter ablation for PsAF is the paradoxical relationship between extensive ablation and limited clinical benefit. Optimizing the balance between ablation extent and clinical efficacy remains critically important. Previous studies [4], [5], [6], [7]suggest that adjunctive strategies beyond PVI—such as linear ablation, complex fractionated atrial electrogram (CFAE) ablation, or Marshall vein ethanolization—may increase procedural success rates. However, major trials including CAPLA, OASIS, MAGIC-AF, Stable SR-II, CHASE-AF, and STAR AF II [11], [12], [13], [14], [15], [16], [22], [23]demonstrate significant limitations of this approach: Despite expanding the anatomical scope of ablation, these combined strategies paradoxically fail to significantly improve long-term clinical outcomes. Excessive ablation may even induce iatrogenic atrial arrhythmias. The CAVAC-AF trial [10]further demonstrated that while adjunctive superior vena cava isolation extended the anatomical ablation scope beyond PVI, it significantly increased risks of phrenic nerve palsy (20.8% vs. 6.0%, *P* = 0.003) and sinoatrial node injury (18.8% vs. 0%, *P* = 0.001), without improving 12-month freedom from atrial arrhythmia (62.9% vs. 72.0%, *P* = 0.41). This paradox underscores how traditional “one-size-fits-all” extended ablation strategies may neglect patient heterogeneity—particularly individual variations in arrhythmogenic substrate. Consequently, contemporary AF ablation research has shifted toward achieving precision-based reduction within procedural augmentation: identifying core arrhythmia mechanisms to avoid nonessential ablation and enable individualized, simplified therapy. The FLOW-AF study [17]identified extrapulmonary trigger foci in 60% of patients with non-paroxysmal AF using electrographic flow mapping. However, empirically extending the ablation lesion set (e.g., routine linear ablation) failed to confer prognostic benefit overall. Subgroup analysis demonstrated that the efficacy of PVI alone in PsAF varied, with its effectiveness dependent on the presence of driver (maintainer) (quantified by CFAE). [24]. The observed 73.9% success rate in the PVI cohort may reflect ibutilide-facilitated identification of patients with preserved atrial substrate, enabling maintained therapeutic efficacy through precision-guided ablation reduction.

### Ibutilide’s exploration from therapy to diagnosis in ablation strategies

This study repurposes ibutilide from therapy to diagnosis, establishing an intraoperative “drug-to-ablation” feedback strategy: by blocking IKr, prolonging the action potential and increasing dispersion of refractoriness, it may unmask latent micro-reentry and focal drivers [13], enabling precise ablation while sparing non-arrhythmogenic tissue. Studies show that intra-operative AF termination predicts lower late recurrence [25], when AF persists, ablation of dispersed electrograms or rotors, or extra-PV lesions tailored by sex and atrial size per TAILORED-AF [19], can be applied, with women deriving greater benefit—consistent with our finding of female sex as an independent recurrence predictor, yet requiring further validation. LVZ prevalence was lower in the PVI group than in the linear-ablation group (4.7% vs. 29.5%), suggesting it helps identify critical targets, conditional on future drug-free controlled studies.

Although ibutilide-response subtypes did not significantly guide strategy selection, the 73.9% sinus-rhythm rate with PVI alone suggests the drug may enable flag a “PVI-responsive” phenotype, allowing unnecessary linear lesions to be safely omitted. This agrees with CHASE-AF22, Singh [26] and Liu [6], who indicated that AF termination probably serve as a procedural endpoint. Within the PVI+linear cohort, the acute termination pattern—especially the need for DC cardioversion—carried distinct prognostic weight, presumably reflecting left-atrial fibrosis (LVZ). Future work should integrate high-resolution mapping and machine-learning risk models to advance phenotype-directed therapy.

### Dynamic ablation strategy enables identification of AF recurrence risk factors

Recurrence following catheter ablation remains a significant clinical challenge. Identifying risk factors for recurrence is crucial for guiding treatment strategies. Zink et al. [27] identified coronary heart disease (HR 1.85, 95%CI 1.20–2.86; *P* = 0.005), intraoperative DC (HR 1.78, 95%CI 1.26–2.49; *P* = 0.001), and elderly female (HR 1.01, 95%CI 1.00-1.01; *P* = 0.04) as factors associated with recurrence. Aguiar-Neves et al. [28 ]found that female were more prone to late recurrence. Matsunaga-Lee et al. [29] confirmed that longer AF duration increases the recurrence risk. Furthermore, the National Registry Study of Denmark [30] demonstrated that early ablation was significantly associated with a reduced risk of AF recurrence compared to delayed ablation. Additionally, patients undergoing early ablation had a significantly lower associated risk of heart failure, ischemic stroke, or death, consistent with findings from the STAR AF II sub-study [31]. In a study of patients with dilated cardiomyopathy and concomitant AF, Siow et al. [20] identified LA diameter as a predictive factor for AF recurrence after catheter ablation.The meta-analysis by Li et al. [32] demonstrated that diabetes mellitus, reduced left ventricular ejection fraction, female, advanced age, longer AF duration, elevated high-sensitivity C-reactive protein levels, LA enlargement, PsAF, and lack of exercise rehabilitation increased the risk of recurrence. In the present study, following the identification of “PVI-sensitive patients” using an ibutilide-guided dynamic ablation strategy, multivariate Cox regression analysis of patients in the PVI+linear ablation group identified female, larger LA diameter, longer AF duration, acute termination pattern as independent predictors of recurrence after catheter ablation.Therefore, using ibutilide to help us effectively identify patients with “PVI-sensitive” PsAF, combined with the analysis of risk factors related to recurrence, is expected to achieve “precise subtraction” while increasing the AF-free survival rate.

### Study limitations

Several limitations warrant acknowledgment. First, this single-center retrospective study carries an inherent risk of selection bias. Validation of long-term efficacy requires future multicenter randomized controlled trials with larger cohorts. Second, voltage mapping performed during AF) may yield artificially low measurements due to functional (rather than structural) abnormalities, potentially compromising accurate substrate characterization. Finally, follow-up relied on intermittent rhythm monitoring (24-hour Holter or serial ECGs) rather than continuous methods (transtelephonic monitoring or implantable loop recorders). Consequently, non-sustained or asymptomatic AF episodes may have been undetected, potentially leading to underestimation of true recurrence rates. In addition, this study did not include a control group without ibutilide administration, which induces a certain bias.

## Conclusion

The use of ibutilide may help identify “PVI‑sensitive” atrial fibrillation, probably avoiding excessive ablation while maintaining procedural success rates. Furthermore, this study suggested female, LA enlargement, prolonged AF duration, and specific acute AF termination patterns as independent predictors of AF recurrence after catheter ablation.

## Data Availability

The data that support the findings of this study are available from the corresponding author, upon reasonable request.

## Abbreviations

AF: Atrial fibrillation
AFL: Atrial flutter
AT: Atrial tachycardia
LA: Left atrium
SR: Sinus rhythm
PVI: Pulmonary vein isolation
LVZs: Low-voltage areas
DC: Direct-electrical cardioversion
CFAE: Complex fragmented atrial potential
